# Role of the mannose receptor in phagocytosis of *Enterococcus faecalis* strain EC-12 by antigen-presenting cells

**DOI:** 10.1002/mbo3.99

**Published:** 2013-06-26

**Authors:** Takeshi Tsuruta, Ryo Inoue, Takayuki Nagino, Ryoichiro Nishibayashi, Yuko Makioka, Kazunari Ushida

**Affiliations:** Laboratory of Animal Science, Kyoto Prefectural University1-5 Shimogamohangityou, Sakyo-ku, Kyoto, Japan

**Keywords:** Antigen-presenting cell, EC-12, LAB, macrophage, mannose receptor, phagocytosis

## Abstract

The aim of this study was to clarify the phagocytic mechanisms of a heat-killed cell preparation of *Enterococcus faecalis* strain EC-12 (EC-12) by antigen-presenting cells (APCs). Fluorescein isothiocyanate (FITC)-labeled EC-12 was cocultured with peritoneal macrophage and the amount of EC-12 phagocytosed by peritoneal macrophages was measured using a microplate fluorometer. Peritoneal macrophages from toll-like receptor (TLR)2-, TLR7-, and MyD88-deficient knockout (KO) mice exhibited similar levels of EC-12 phagocytosis to those from wild-type mice. Similarly, dectin-1 neutralization of peritoneal macrophages had no effect on EC-12 phagocytosis. However, blockade of the mannose receptor (MR) significantly decreased the amount of EC-12 phagocytosed by peritoneal macrophages; the same effect was observed in bone marrow-derived macrophages and dendritic cells. Our findings suggest that MR plays a major role in EC-12 phagocytosis by the APCs.

This aim of this study was to clarify the phagocytic mechanisms of a heat-killed cell preparation of *Enterococcus faecalis* strain EC-12 (EC-12) by antigen-presenting cells (APCs). Our findings suggest that mannose receptor (MR) plays a major role in EC-12 phagocytosis by the APCs.

## Introduction

Lactic acid bacteria (LAB) are regarded as symbiotic microorganisms that confer beneficial effects, such as preventing tumor development, limiting allergic reactions, and fighting infection (Reid et al. 1990; Cross et al. 2001; Asahara et al. 2004; Lee et al. 2004; Inoue et al. 2007). Therefore, they are used widely in the care of humans and livestock animals.

A part of ingested LAB are taken up from M cells of the gastrointestinal tract and phagocytosed by antigen-presenting cells (APCs) such as macrophages present within Peyer's patches and the lamina propria (Jang et al. 2004; Galdeano and Perdigon 2006; Inoue et al. 2013). The macrophages activated by LAB secrete cytokines such as IL-12, which plays a pivotal role in inducing the T helper 1-dominant immune response and enhancing cellular immunity (Trinchier 1995).

Our previous study demonstrated that nucleic acids, especially RNA, from heat-killed cell preparations of *Enterococcus faecalis* strain EC-12 (EC-12) induced IL-12 production by the murine macrophage-like cell line J774.1. This effect was mediated by toll-like receptor (TLR) 7 (Inoue et al. 2011). Because TLR7 is expressed in endosomes but not cell membranes, EC-12 phagocytosis by APCs is an important event for the initiation of probiotic-like effects derived by EC-12 (Kuramoto et al. 2004; Tsuruta et al. 2009; Yoshikawa et al. 2009).

TLRs contribute to phagocytosis of pathogenic bacteria such as *Listeria monocytogenes* (TLR2), *Esherichia coli* (TLR2/4), and *Salmonella typhimurium* (TLR2/4) by macrophages (Blander and Medzhitov 2004; Shen et al. 2010). Dectin-1 also plays a role in phagocytosis of fungi, pathogenic bacteria, and plants through the recognition of particulate and soluble β-glucan (Rothfuchs et al. 2007).

The mannose receptor (MR) is known to recognize mannose, fucose, and N-acetylglucosamine in a Ca^2+^-dependent manner and is expressed by most macrophage populations (Taylor et al. 2005). MR-dependent phagocytosis of *Mycobacterium tuberculosis*,* Pseudomonas aeruginosa,* and *Candida albicans* were reported previously (Gordon and Read 2002; Kerrigan and Brown 2009).

Although the roles of these receptors in phagocytosis of pathogenic microorganisms are clear, their contribution to phagocytosis of beneficial microorganisms such as LAB and EC-12 has not been fully elucidated. Accordingly, we investigated the role of TLRs, dectin-1, and MR in EC-12 phagocytosis by macrophages residing in the peritoneum. To this end, we also explored the effect of MR blockade on phagocytosis by bone marrow-derived macrophages (BMDMs) and dendritic cells (BMDCs), as the peritoneal population of macrophages is suggested to be functionally and phenotypically heterogenous (Bogdan and Schleicher 2006; Pollard 2009; Ghosn et al. 2010).

## MATERIALS AND METHODS

### Experiment 1

#### Animals

Wild-type (WT), TLR2-, TLR7-, and MyD88-deficient knockout (KO) mice that had been bred on a C57BL/6 background were used in this study. Three 10-week-old mice with each of these genetic makeups were used for the experiment. The WT mice were purchased from Japan SLC (Shizuoka, Japan). The TLR2-, TLR7-, and MyD88-KO mice were originally obtained from Oriental Bioservice (Kyoto, Japan) and then bred in our pathogen-free animal facility. All mice were allowed ad libitum access to normal rodent chow (Lab MR stock; Nihon Nosan Kogyo, Tokyo, Japan) and water. All of the animal experiments were performed in accordance with the guidelines for studies on laboratory animals approved by the Kyoto Prefectural University Experimental Animal Committee.

#### Fluorescent labeling of EC-12

EC-12, a commercial product of the cell preparation of *E. faecalis* strain EC-12, was a kind gift from Mr. Takumi Watanabe of the Combi Corporation (Saitama, Japan). The preparation of heat-killed bacteria was in the form of a dried powder. One hundred milligrams of EC-12 were suspended in 20 mL of 16 nmol L^−1^ fluorescein isothiocyanate (FITC) in carbonate buffer (50 mmol L^−1^ Na_2_CO_3_, 50 mmol L^−1^ NaHCO_3_, pH 9.6). The suspension was incubated for an hour at 37°C. After washing the samples with phosphate-buffered saline (PBS; 20,400*g*, 4°C, 5 min), the bacterial pellet was suspended with culture media (RPMI medium [Nakalai tesque, Kyoto, Japan]) containing 10% fetal bovine serum, penicillin (100 U mL^−1^, Sigma-Aldrich Japan, Tokyo, Japan), and streptomycin (100 μg mL^−1^, Sigma-Aldrich Japan) at a concentration of 2 mg mL^−1^.

#### Macrophage preparation

Each mouse was anesthetized using an intraperitoneal injection of 30 μL of sodium pentobarbital (Schering-Plough, Osaka, Japan). This was followed by exsanguination. After euthanasia, 5 mL cold PBS was applied to the peritoneal cavity through a syringe. The abdominal site was gently kneaded for 3 min. The peritoneal fluid containing peritoneal macrophages was collected through the syringe and centrifuged for 3 min (4°C, 400*g*). After removal of the supernatant, the cells were suspended in the above-mentioned culture media. The cell suspensions were stained with trypan blue and counted using a TC10 automated cell counter (Bio-Rad, Richmond, CA).

For the quantitative evaluation, the peritoneal macrophages were cultured in 96-well flat-bottom culture plates (Orange Scientific, Tokyo, Japan) with 100 μL of culture media at a concentration of 1 × 10^5^ cells per well. The cells were preincubated for 4 h at 37°C in a humidified 5% CO_2_ incubator.

For the image analysis, the peritoneal macrophages were cultured on culture slides (Beckton Dickinson, Tokyo, Japan) with 500 μL of culture media at a concentration of 5 × 10^5^ cells per well and preincubated as described above.

#### Quantitative evaluation of EC-12 phagocytosis

Ten microliters of FITC-labeled EC-12 cells (2 mg mL^−1^) were added to each well of the 96-well culture plates containing the preincubated peritoneal macrophages from the WT, TLR2-, TLR7-, and MyD88-KO mice. The cocultures were stored in a humidified 5% CO_2_ incubator. Samples were incubated for 1 or 2 h, as our preliminary experiments indicated that almost all peritoneal macrophages completely phagocytose EC-12 within 2 h after exposure. Each experiment was performed in triplicate.

After incubation, the culture media were removed. The remaining nonphagocytosed EC-12 cells were washed away with PBS. Then, 100 μL of cold methanol was added to each well and the cells were fixed for 1 min. After washing the samples with PBS, 100 μL of 4′, 6-diamidino-2-phenylindole (DAPI) solution in PBS (0.5 μg mL^−1^) was added to each well. The levels of FITC and DAPI fluorescence were measured using a microplate fluorometer (ARVO™ X3, PerkinElmer Japan, Yokohama, Japan). Measurements of FITC fluorescence were normalized to DAPI levels. The normalized value was centuplicated and expressed as arbitrary units (AUs).

#### Image analysis

Fifty microliters of the FITC-labeled EC-12 (2 mg mL^−1^) suspension were added to each well of the culture slides containing the preincubated peritoneal macrophages. Cells were incubated for 1 or 2 h. After incubation, the culture media were removed from each well and the cells were washed and fixed as above. After fixation, each well was washed with PBS and mounted with Vectashield Mounting Medium with DAPI (Vector Laboratories, Burlingame, CA). The glass slide preparations were examined under a fluorescent microscope (E600; Nikon, Tokyo, Japan). All images were photographed using a Ds-Fi1c digital camera (Nikon) attached to E600.

#### Dectin-1 neutralization and MR blockade

Dectin-1 neutralization and MR blockade, respectively, were achieved by adding 100 μL of culture media containing anti-dectin-1 antibody (0.1 mg mL^−1^, Invivogen, CA) or yeast-derived mannan (10 mg mL^−1^, Nakalai tesque) to the media of the wells containing preincubated peritoneal macrophages from the WT mice (Schulert and Allen 2006; Yadav and Schorey 2006). Ten microliters of FITC-labeled EC-12 (2 mg mL^−1^) were added to the solution; the cells were incubated for 30 min. The same volume of a FITC-labeled preparation of heat-killed *C. albicans* (HKCA: 1 × 10^7^ cells mL^−1^) was used as a positive control for dectin-1-mediated phagocytosis. For MR-mediated phagocytosis, the same volume of a FITC-labeled zymosan (1 × 10^7^ cells mL^−1^) and green fluorescent silica beads (250 μg mL^−1^; Corefront Co., Ltd., Tokyo, Japan) was used as a positive control and negative control, respectively. The quantitative evaluations and image analysis of labeled EC-12 phagocytosis were performed as described above.

#### Statistical analysis

Data are presented as mean values with standard variations. The amount of EC-12 phagocytosed by peritoneal macrophages from the WT, TLR2-, TLR7-, and MyD88-KO mice was analyzed using one-way analysis of variance (ANOVA) (the factor of variation was the genetic background). When significant differences were detected, a Tukey–Kramer post hoc comparison was performed. The unpaired Student's *t*-test was used to test the effects of dectin-1 neutralization and MR blockade on EC-12 phagocytosis by peritoneal macrophages. Differences among means were considered as significant at *P *< 0.05. All data were analyzed using JMP 8.0 software (JMP, Tokyo, Japan).

### Experiment 2

#### Preparation of BMDMs and BMDCs

BMDMs and BMDCs were obtained from the WT mice as described by Hill et al. (2003). In brief, BM cells were extracted from the femurs of adult mice and washed with the same type of culture media used for experiment 1. To induce macrophage differentiation, the BM cells were cultured in the presence of 100 ng mL^−1^ of recombinant mouse macrophage colony-stimulating factor (PeproTech EC, London, U.K.). The BM cells were cultured in the presence of 100 ng mL^−1^ of recombinant granulocyte/macrophage colony-stimulating factor (10 ng mL^−1^, PeproTech EC) to induce differentiation to myeloid DCs. Fresh media were added every 2–3 days, and the cells were harvested after 7 days of incubation. The macrophage preparation was >90% CD11b+ and >80% F4/80+ cells. The DC preparation comprised >90% CD11c+ B220− cells. The cells were resuspended in culture media, seeded at a concentration of 1 × 10^5^ cells per well in a 96-well culture plate, and incubated for 24 h prior to the experiment.

#### MR blockade and quantitative evaluation of EC-12 phagocytosis

MR blockade, quantitative evaluation of EC-12 phagocytosis, and statistical analysis were all performed as in experiment 1.

## Results

### EC-12 phagocytosis by peritoneal macrophages is independent of TLR2, TLR7, and MyD88 signaling

**Figure 1:**
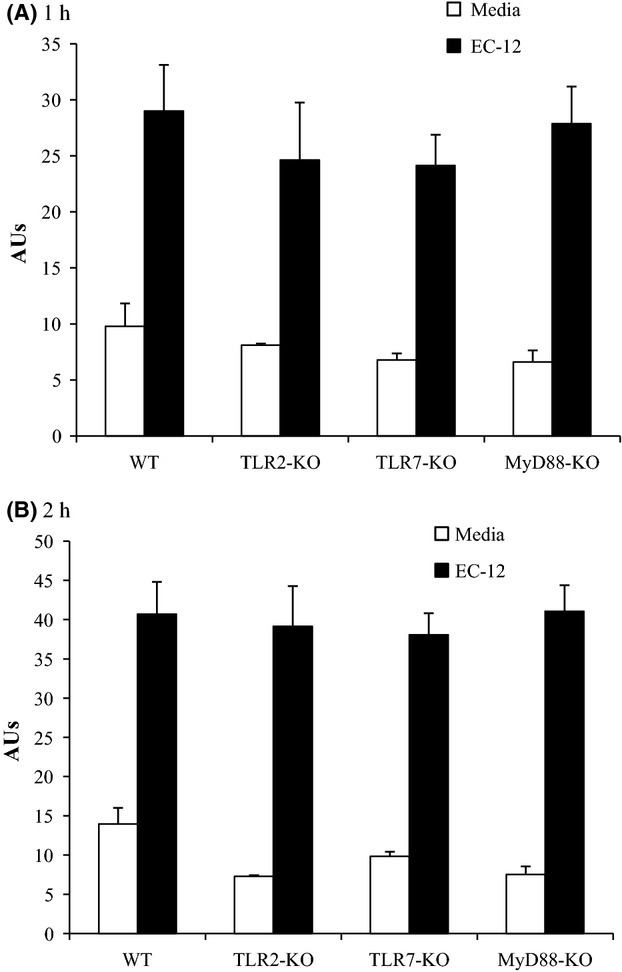
The amount of EC-12 phagocytosed by peritoneal macrophages from WT, TLR2-, TLR7-, and MyD88-KO mice. The peritoneal macrophages preincubated in a 96-well microplate were cocultured with FITC-labeled EC-12 for 1 (A) or 2 h (B). After nuclear staining with DAPI, the levels of DAPI and FITC fluorescence were measured using a microplate fluorometer. Measurements of FITC fluorescence were normalized to DAPI levels. The normalized value was centuplicated and expressed as arbitrary units (AUs). Error bars indicate the standard deviation of the mean. WT, wild-type; TLR, toll-like receptor; KO, knockout; FITC, fluorescein isothiocyanate; DAPI, 4′, 6-diamidino-2-phenylindole.

**Figure 2:**
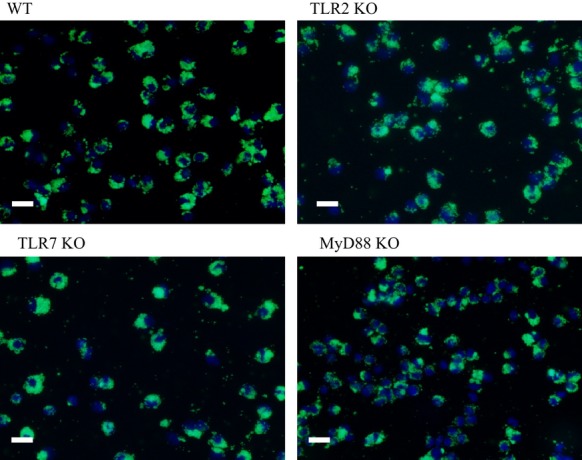
Image analysis of EC-12 phagocytosis by peritoneal macrophages from WT, TLR2-, TLR7-, and MyD88-KO mice. The peritoneal macrophages preincubated in a culture slide were cocultured with FITC-labeled EC-12 for 2 h. After mounting with DAPI medium, the glass slide preparations were assessed under a fluorescent microscope. All images were photographed using a Ds-Fi1c digital camera attached to E600. A representative image is shown. Bar represents 20 μm. WT, wild-type; TLR, toll-like receptor; KO, knockout; FITC, fluorescein isothiocyanate; DAPI, 4′, 6-diamidino-2-phenylindole.

The peritoneal macrophages from each strain of mice phagocytosed EC-12 cells (Figs. 1, 2). After 1 h of incubation, no significant difference was observed in the amount of EC-12 phagocytosed among all mouse strains investigated (WT: 28.99 ± 4.11 AUs, TLR2-KO: 24.63 ± 5.12 AUs, TLR7-KO: 24.14 ± 2.74 AUs, MyD88-KO: 27.87 ± 3.31 AUs, Fig. 1A). The amount of EC-12 phagocytosed was markedly increased after 2 h compared with after 1 h of incubation. Again, each mouse strain exhibited similar levels of EC-12 phagocytosis by peritoneal macrophages (WT: 40.69 ± 3.78 AUs, TLR2-KO: 39.17 ± 6.44 AUs, TLR7-KO:38.07 ± 3.46 AUs, MyD88-KO: 41.07 ± 2.12 AUs, Fig. 1B). The observations made during microscopic examinations supported the results obtained by quantitative analysis (Fig. 2).

### EC-12 phagocytosis by peritoneal macrophages occurred independent of dectin-1 signaling

**Figure 3:**
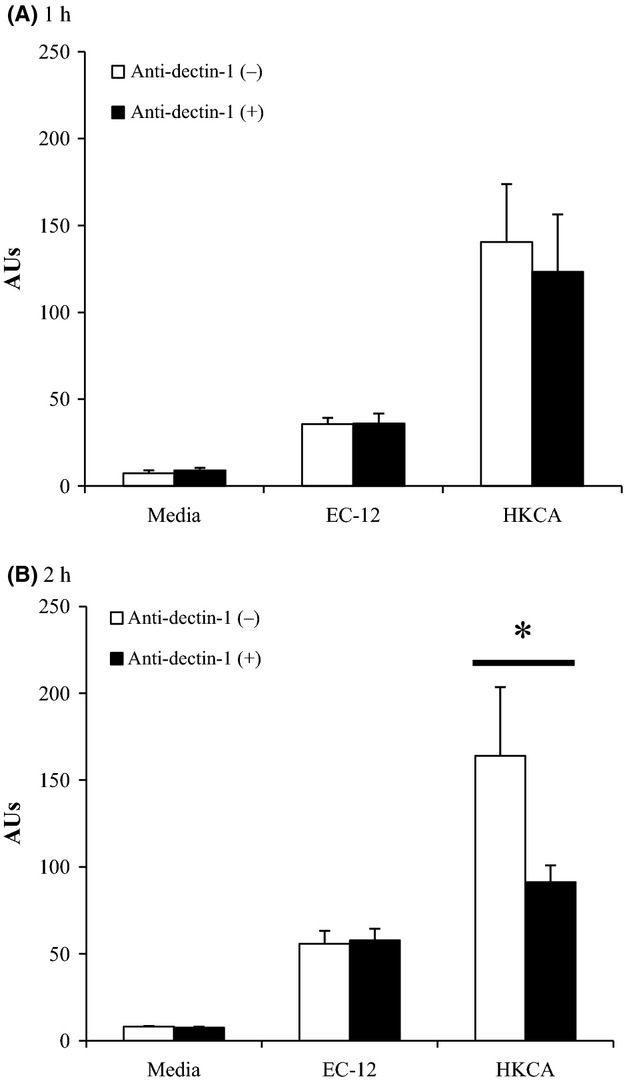
The effect of dectin-1 neutralization on EC-12 phagocytosis by WT peritoneal macrophages. Dectin-1 on peritoneal macrophages was neutralized with anti-dectin-1 antibody, and the macrophages were cocultured with FITC-labeled EC-12 or HKCA for 1 (A) or 2 h (B). The levels of macrophage-mediated EC-12 and HKCA phagocytosis are shown in AUs. (*) A statistically significant difference was considered at *P *< 0.05. Error bars indicate the standard deviation of the mean. WT, wild-type; FITC, fluorescein isothiocyanate; HKCA, heat-killed *Candida albicans*; AUs, arbitrary units.

The amount of EC-12 and HKCA phagocytosed by WT peritoneal macrophages with or without dectin-1 neutralization is shown in Figure 3A (1 h incubation) and Figure 3B (2 h incubation). The amount of HKCA phagocytosed by the WT peritoneal macrophages tended to be lower among dectin-1-neutralized cells after 1 h of incubation and was significantly lower among these cells after 2 h of incubation (Fig. 3B). On the other hand, no difference was observed between dectin-1-neutralized and control cells in terms of the amount of EC-12 phagocytosed (Fig. 3A and B).

### MR plays a major role in EC-12 phagocytosis by peritoneal macrophages, BMDMs, and BMDCs

**Figure 4:**
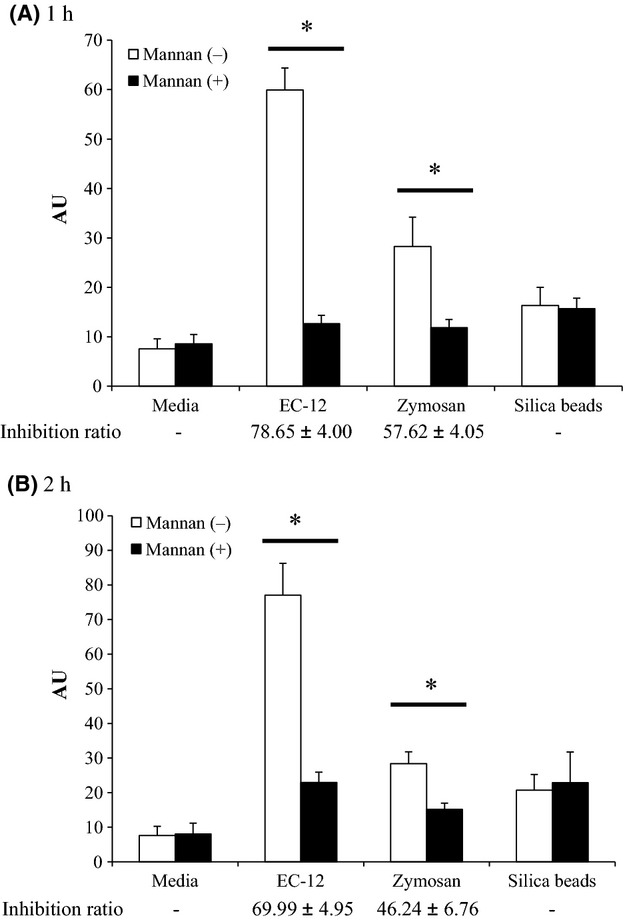
The effect of MR blockade on EC-12 phagocytosis by WT peritoneal macrophages. The peritoneal macrophages were treated with mannan and cocultured with FITC-labeled EC-12, zymosan or silica beads for 1 (A) or 2 h (B). The levels of macrophage-mediated EC-12, zymosan and silica beads phagocytosis are shown in AUs. The rate of phagocytosis inhibition induced by the MR blockade is shown under the horizontal axis of each graph. (*) A statistically significant difference was considered at *P *< 0.05. Error bars indicate the standard deviation of the mean. WT, wild-type; FITC, fluorescein isothiocyanate; AUs, arbitrary units; MR, mannose receptor.

**Figure 5:**
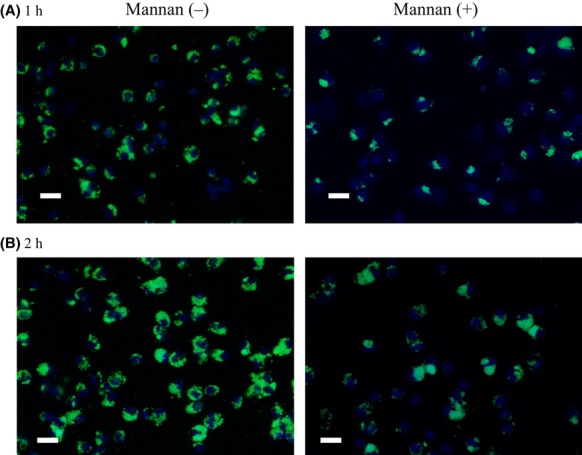
Image analysis of EC-12 phagocytosis by peritoneal macrophages treated with or without mannan. The peritoneal macrophages were treated with mannan for the MR blockade and cocultured with FITC-labeled EC-12 for 1 (A) or 2 h (B). After mounting with DAPI medium, the glass slide preparations were assessed under a fluorescent microscope. All images were photographed using a Ds-Fi1c digital camera attached to E600. A representative image is shown. Bar represents 20 μm. MR, mannose receptor; FITC, fluorescein isothiocyanate; DAPI, 4′, 6-diamidino-2-phenylindole.

The amount of EC-12, zymosan, and silica beads phagocytosed by the WT peritoneal macrophages was examined with or without the MR blockade (Fig. 4A and B). The amount of silica beads phagocytosed by the peritoneal macrophages was not changed by the MR blockade in the 1- and 2-h incubation groups (1 h incubation: mannan (−), 16.32 ± 3.67 AUs; mannan (+), 15.68 ± 2.14 AUs; 2 h incubation: mannan (−), 20.69 ± 22.89 AUs; mannan (+): 22.89 ± 8.83 AUs). The amount of zymosan phagocytosed by the peritoneal macrophages was significantly decreased by the MR blockade in the 1- and 2-h incubation groups (1 h incubation: mannan (−), 28.26 ± 5.94 AUs; mannan (+), 11.85 ± 1.64 AUs; 2 h incubation: mannan (−), 28.35 ± 3.41 AUs; mannan (+): 15.16 ± 1.78 AUs). Similarly, the amount of EC-12 phagocytosed by the peritoneal macrophages was significantly decreased by the MR blockade, regardless of the duration of incubation (1 h incubation: mannan (−): 59.89 ± 4.47 AUs; mannan (+): 12.68 ± 1.66 AUs; 2 h incubation: mannan (−): 77.05 ± 9.2 AUs; mannan (+): 22.91 ± 3.01 AUs). The rate of EC-12 phagocytosis inhibition achieved by the MR blockade was 78.65 ± 4.00% and 69.99 ± 4.95% in the 1-h and 2-h incubation groups, respectively. Microscopic observations supported these findings (Fig. 5).

**Figure 6:**
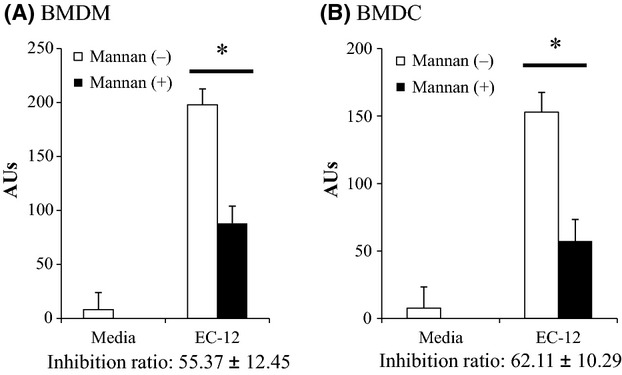
The effect of MR blockade on EC-12 phagocytosis by BMDMs (A) and BMDCs (B). The cells were treated with mannan for MR blockade and cocultured with FITC-labeled EC-12 for 1 or 2 h. The level of macrophage-mediated EC-12 phagocytosis is shown in AUs. The rate of EC-12 phagocytosis inhibition achieved by the MR blockade was calculated as in Figure 4 and displayed under the horizontal axis of each graph. (*) A statistically significant difference was considered at *P *< 0.05. Error bars indicate the standard deviation of the mean. MR, mannose receptor; BMDMs, bone marrow-derived macrophages; BMDCs, bone marrow dendritic cells; FITC, fluorescein isothiocyanate.

The amount of EC-12 phagocytosed by BMDMs and BMDCs was also significantly decreased by the MR blockade (Fig. 6). The rates of EC-12 phagocytosis inhibition by the MR blockade were 55.37 ± 12.45% and 62.11 ± 10.29% for the 1-h and 2-h incubation groups, respectively.

## Discussion

While the mechanism of phagocytosis of pathogenic microorganisms by APCs has been well clarified, few studies have assessed the type of receptors involved in LAB phagocytosis.

In experiment 1, we examined the role of TLR2, TLR7, and MyD88 in EC-12 phagocytosis by peritoneal macrophages. TLR2 binds peptidoglycan (PGN), a major component of the LAB cell wall. We previously reported that EC-12 RNA binds to TLR7. However, the levels of EC-12 phagocytosis by the peritoneal macrophages obtained from the TLR2-, TLR7-, and MyD88-KO mice were similar to those by the peritoneal macrophages obtained from the WT mice (Figs. 1, 2). Similar results were reported by Ichikawa et al. (2012); no significant difference was observed in the amount of *Lactobacillus paracasei* KW3110 phagocytosed by peritoneal macrophages from WT, TLR2, TLR4, or MyD88-KO mice.

MR but not dectin-1 appears to contribute strongly to EC-12 phagocytosis. The amount of EC-12 phagocytosed by the peritoneal macrophages was not affected by dectin-1 neutralization but was reduced (~70%) in the presence of an MR blockade. Similar reduction rate of the phagocytosis was observed by an MR blockade in viable EC-12 (Data not shown). MR-mediated phagocytosis of microorganisms is thought to involve the recognition of mannose, fucose, and N-acetylglucosamine. LAB species, including EC-12, contain these sugar residues in their PGN and exopolysaccharides (EPSs). In addition to PGN and EPSs, Polotsky et al. (1996) reported that lipoteichoic acid residues from *E. faecalis* strain Kiel 27738 have high affinity for human recombinant MR.

The role of MR in EC-12 phagocytosis was also demonstrated in studies of BMDMs and BMDCs (experiment 2). EC-12 phagocytosis was markedly inhibited by the MR blockade in the BMDM and BMDC cultures (Fig. 6). The rate of EC-12 phagocytosis inhibition was slightly different among the cell types. However, in all cell types used, a large part of EC-12 phagocytosis seemed to depend on MR (Fig. 6).

In conclusion, this study suggests that the MyD88 signaling pathway, including the TLR2 and TLR7 cascades, and dectin-1, might not contribute or contribute less, if any, to EC-12 phagocytosis by APCs. MR is thought to be the major receptor for EC-12 phagocytosis by APCs. We have confirmed in the preliminary experiment that the MR blockade of macrophage-like cell line J774.1 affected phagocytosis of 11 strains of LAB used in the article by Inoue et al. (2011). The MR blockade was most effective in inhibiting phagocytosis of *E. faecalis* ATCC19433 (85.43 ± 4.96% inhibition), and least effective in inhibiting phagocytosis of *L. acidophilus* JCM1134 (31.28 ± 22.80%). Therefore, MR might not always be a major receptor for phagocytosis of all LAB species/strains by APCs, but should be the commonly used receptor for phagocytosis of most LAB strains. Goh et al. (2005) reported that the amount of EPSs produced by LAB species and their sugar composition could be different among LAB strains even in same species. Furthermore, EPS production by one single strain of LAB is affected by culture conditions such as temperature and pH (Gamar-Nourani et al. 1998). These facts imply that, if an efficient immunostimulation is expected, the amount of MR ligand could be considered as one factor for screening and preparation of LAB strain.

## Conflict of Interest

None declared.
